# Prognostic implications of PD-L1 expression in patients with angiosarcoma

**DOI:** 10.2144/fsoa-2020-0211

**Published:** 2021-03-02

**Authors:** Jii Bum Lee, Beung-Chul Ahn, Seung Hyun Kim, Young Han Lee, Jung Woo Han, Min Kyung Jeon, Soo Hee Kim, Hyo Song Kim

**Affiliations:** 1 Department of Internal Medicine, Division of Medical Oncology, Yonsei Cancer Center, Yonsei University College of Medicine, Seoul, Korea; 2 Department of Orthopedic Surgery, Yonsei University College of Medicine, Seoul, Korea; 3 Department of Radiology, Yonsei University College of Medicine, Seoul, Korea; 4 Department of Pediatric Hemato-Oncology, Yonsei University College of Medicine, Seoul, Korea; 5 Pathology Center, Seegene Medical Foundation, Seoul, Korea

**Keywords:** angiosarcoma, chemotherapy, overall survival, PD-L1, prognosis

## Abstract

**Aim:**

There are limited data on the feasibility of programmed death ligand-1 (PD-L1) expression as a prognostic biomarker in metastatic angiosarcoma.

**Patients & methods:**

We retrospectively collected and analyzed the data on PD-L1 expression in 70 angiosarcoma patients who were diagnosed at our center between 2005 and 2019.

**Results:**

Thirteen (19%) patients had PD-L1 expression. Metastatic angiosarcoma patients who were PD-L1-negative (n = 24) showed longer median progression-free survival (4.9 vs 1.6 months; p = 0.04) and median overall survival (OS; 10.9 vs 5.4 months; p = 0.01) than those who were PD-L1-positive (n = 4). PD-L1 status proved to be a significant factor for OS.

**Conclusion:**

Metastatic angiosarcoma patients with PD-L1 expression showed shorter survival. PD-L1 status is an independent prognostic factor for OS in metastatic angiosarcoma patients.

## Lay abstract

Angiosarcoma, a rare subtype of sarcoma, is highly aggressive and has poor prognosis. The majority of patients are diagnosed at advanced and metastatic stages, and treatment options for these subsets of patients are limited to cytotoxic chemotherapy. Validation of prognostic markers including PD-L1 expression remain to be fully carried out in metastatic angiosarcoma. In our study, we assessed the PD-L1 expression in metastatic angiosarcoma and its role as a prognostic biomarker.

Angiosarcoma, which originates from lymphatic endothelial cells of the blood vessels, is a rare subtype of soft tissue sarcoma (STS) [1]. STS accounts for less than 1% of all adult malignancies, and angiosarcoma accounts for less than 1% of all STS subtypes [2,3]. Angiosarcoma mainly affects adults and elderly patients and can manifest in any anatomic site in any part of the body, including cutaneous lesions, which account for 60% of all cases [4,5]. Angiosarcoma is highly aggressive and is associated with poor prognosis, with a median overall survival (mOS) of 6–16 months [6]. Among patients with angiosarcoma, 20–30% of patients are initially diagnosed in the metastatic setting, and approximately 50% of the patients with localized disease experience disease progression [7–9].

A few cytotoxic chemotherapeutic drugs are effective against metastatic angiosarcoma, such as anthracycline, taxane and pazopanib. Anthracycline-based chemotherapy, in combination with ifosfamide, as the first-line treatment for angiosarcoma showed similar outcomes to those who had received first-line treatment against other STSs, with improved progression-free survival (PFS) [10]. In contrast with other subtypes of STS, angiosarcoma is sensitive to taxane as the first- or second-line treatment [11–13]. Pazopanib, a multitarget tyrosine kinase inhibitor, as second- or later-line therapy also showed improvement in PFS, but no benefit in overall survival (OS) [14]. The rarity and poor prognosis of angiosarcoma further limit prospective trials necessary for the development of treatment options.

Recently, anti-programmed death-1 (anti-PD-1) inhibitors such as pembrolizumab and nivolumab showed the modest clinical activity in the subset of advanced STS [15,16]. However, angiosarcoma patients were not enrolled in the SARC028 trial, and only three patients were evaluated in the nivolumab and ipilimumab group in the Alliance A091401 trial. There is limited evidence on the clinical implications of PD-L1 in angiosarcoma. Thus, our study aimed to investigate PD-L1 expression in metastatic angiosarcoma and evaluate its clinical relevance. In addition, we evaluated the feasibility of PD-L1 expression as a prognostic biomarker that could aid in the design of effective STS treatments.

## Patients & methods

### Study design & patient selection

We collected data from 70 angiosarcoma patients diagnosed from 2005 to 2019 in a single center. The key inclusion criteria included: pathologically confirmed angiosarcoma; formalin-fixed, paraffin-embedded (FFPE) tissue blocks available for the examination of PD-L1 expression; and complete medical history records that included last follow-up or death date.

FFPE tissue blocks were collected from surgical or biopsied specimens, which were performed at baseline or after recurrence. All hematoxylin and eosin (H&E)-stained slides were reviewed by one pathologist (SH Kim), and the clinical data were obtained following a review of the medical records. Clinicopathological variables such as age, sex, tumor location, differentiation, status of resection margin, initial presentation of the disease and palliative chemotherapy were retrospectively reviewed. Tumor location was divided into four categories consisting of the head and neck, thorax, abdomen and extremity. Histology included well-differentiated angiosarcoma, spindle cell angiosarcoma, epithelioid angiosarcoma and poorly differentiated angiosarcoma. The 8th edition of the American Joint Committee on Cancer guideline of tumor, node and metastasis (TNM) classification was used for determining cancer stage [17].

### Tissue selection, immunohistochemistry staining & assessment

The H&E-stained slides were examined, and one or two representative FFPE archival blocks were selected for each case. Immunohistochemical staining was performed using PD-L1 monoclonal antibody, Clone 22C3 (Agilent Technologies, CA, USA/Dako, CA, USA) and the DAKO Link 48 system (Agilent, CA, USA). After deparaffinization, heat-induced antigen retrieval was performed using EnVision™ FLEX Target Retrieval Solution, Low pH (Dako Omnis). Positive rate for PD-L1 was scored by an experienced pathologist (SH Kim). We analyzed the combined positive score (CPS) and tumor proportion score of the PD-L1-immunostained slides. Tumor cell positivity includes partial and complete linear membrane staining at any intensity. Immune cell positivity includes cytomembranous expression of lymphocytes and macrophages. CPS was defined as the number of PD-L1-positive cells (tumor cells, macrophages, lymphocytes) divided by the total number of tumor cells and multiplied by 100 [18]. PD-L1 positivity was defined as CPS ≥1.

### Statistical analysis

Statistical analysis was performed using SPSS, version 25 (IBM, IL, USA), and GraphPad Prism 8.0 software (GraphPad Software, Inc., CA, USA). The correlations between the variables were analyzed using the Fisher’s exact test for categorical variables and the sample *t*-test for continuous variables. We used the Kaplan–Meier method for calculating OS and PFS [19]. The overall response rate was calculated as the percentage of patients experiencing a confirmed complete response or partial response as per the Response Evaluation Criteria in Solid Tumors (RECIST) 1.1 guidelines. PFS was defined as the time from the start of first-line palliative chemotherapy to the date of progression or death. OS was defined as the period from the diagnosis of metastatic or recurrent angiosarcoma until the date of the last follow-up or death. Cox regression was used for the multivariate analysis of OS. Statistical analysis was two-sided, with a p-value of less than 0.05 considered statistically significant.

## Results

### Patient characteristics

Table 1 summarizes the clinicopathological characteristics of the 70 angiosarcoma patients according to the PD-L1 status (CPS ≥1). The male-to-female ratio was 1.33, and the median patient age was 61 years (range: 7–86 years). The most common tumor location was the head and neck (n = 28, 40%), followed by the abdomen (n = 18, 26%), thorax (n = 15, 21%) and extremity (n = 9, 13%). Most of the patients were diagnosed with well-differentiated angiosarcoma (n = 29, 41%) and poorly differentiated angiosarcoma (n = 24, 34%). Other histology subtypes included spindle (n = 14, 20%) and epithelioid angiosarcoma (n = 3, 5%). For patients who underwent surgery (n = 41, 59%), most had negative resection margins (n = 43, 61%). Among these patients, six (9%) received adjuvant chemotherapy and 19 (27%) were treated with adjuvant radiotherapy.

**Table 1.** Baseline characteristics of all patients.

Variables		PD-L1 expression			
		Total n = 70 (%)	CPS <1 n = 57 (81%)	CPS ≥1 n = 13 (19%)	p-value
Sex	Male	40 (57%)	35 (87%)	5 (13%)	0.21
	Female	30 (43%)	22 (73%)	8 (27%)	
Median age, years (range)		61 (7–86)	61 (7–86)	59 (25–84)	
Median tumor size, cm (range)		4 (0.3–99)	4 (0.3–99)	5 (0.7–61)	
Tumor location	Head and neck	28 (40%)	21 (75%)	7 (25%)	0.45
	Thorax	15 (21%)	14 (93%)	1 (7%)	
	Abdomen	18 (26%)	14 (78%)	4 (22%)	
	Extremity	9 (13%)	8 (89%)	1 (11%)	
Histology	Well differentiated	29 (41%)	28 (97%)	1 (3%)	0.01
	Spindle	14 (20%)	13 (93%)	1 (7%)	
	Epithelioid	3 (5%)	0 (0%)	3 (100%)	
	Poorly differentiated	24 (34%)	16 (67%)	8 (33%)	
Initial distant metastasis	No	42 (60%)	33 (79%)	9 (21%)	0.54
	Yes	28 (40%)	24 (86%)	4 (14%)	
Surgery	No	29 (41%)	26 (90%)	3 (10%)	0.21
	Yes	41 (59%)	31 (76%)	10 (24%)	
Resection margin^†^	Negative	43 (61%)	36 (84%)	7 (16%)	0.99
	Positive	21 (30%)	18 (86%)	3 (14%)	
Definitive radiotherapy	No	59 (84%)	48 (81%)	11 (19%)	0.99
	Yes	11 (16%)	9 (82%)	2 (18%)	
Adjuvant chemotherapy	No	64 (91%)	52 (81%)	12 (19%)	0.99
	Yes	6 (9%)	5 (63%)	1 (17%)	
Adjuvant radiotherapy	No	51 (73%)	43 (84%)	8 (16%)	0.32
	Yes	19 (27%)	14 (74%)	5 (26%)	
Palliative chemotherapy	No	26 (37%)	20 (77%)	6 (23%)	0.53
	Yes	44 (63%)	37 (84%)	7 (16%)	
Palliative radiotherapy	No	43 (61%)	34 (79%)	9 (21%)	0.75
	Yes	27 (39%)	23 (85%)	4 (15%)	
First-line chemotherapy^‡^	Paclitaxel	31 (44%)	25 (81%)	6 (19%)	0.85
	Pazopanib	3 (5%)	2 (67%)	1 (33%)	
	Others	10 (14%)	8 (80%)	2 (20%)	

† Resection margin data were not available for six patients.

‡ Twenty-six patients (37%) did not receive palliative chemotherapy.

CPS: Combined positive score; PD-L1: Programmed death ligand-1.

Twenty-eight patients (40%) were initially diagnosed with distant metastasis, and 44 metastatic and recurrent patients (67%) received palliative systemic chemotherapy. Most patients received paclitaxel (n = 31, 44%), followed by other regimens (n = 10, 14%) and pazopanib (n = 3, 5%). Other regimens that were administered included adriamycin and ifosfamide (n = 4); ifosfamide (n = 1); doxorubicin and olaparib (n = 1); mesna, doxorubicin, ifosfamide and dacarbazine (n = 1); etoposide, ifosfamide, cisplatin (n = 1); etoposide and cisplatin (n = 1); and vincristine, cyclophosphamide and doxorubicin alternatively followed by the ifosfamide and etoposide regimen (n = 1).

### PD-L1 expression status & clinicopathological features

**Figure 1.**
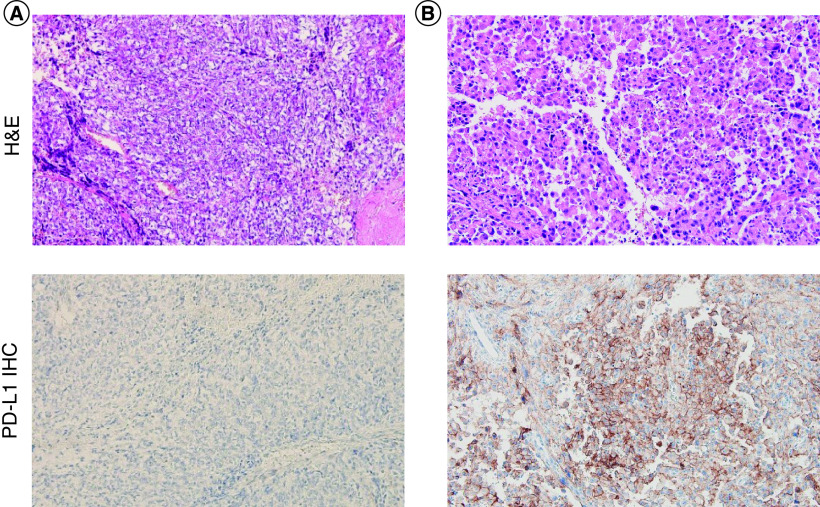
H&E staining and IHC of PD-L1 expression in angiosarcoma. H&E staining (×200) and IHC (×200) of PD-L1 expression in angiosarcoma patients according to **(A)** PD-L1-negative angiosarcoma and **(B)** PD-L1-positive epithelioid angiosarcoma. CPS: Combined positive score; H&E: Hematoxylin and eosin; IHC: Immunohistochemistry; PD-L1: Programmed death ligand-1.

**Figure 2.**
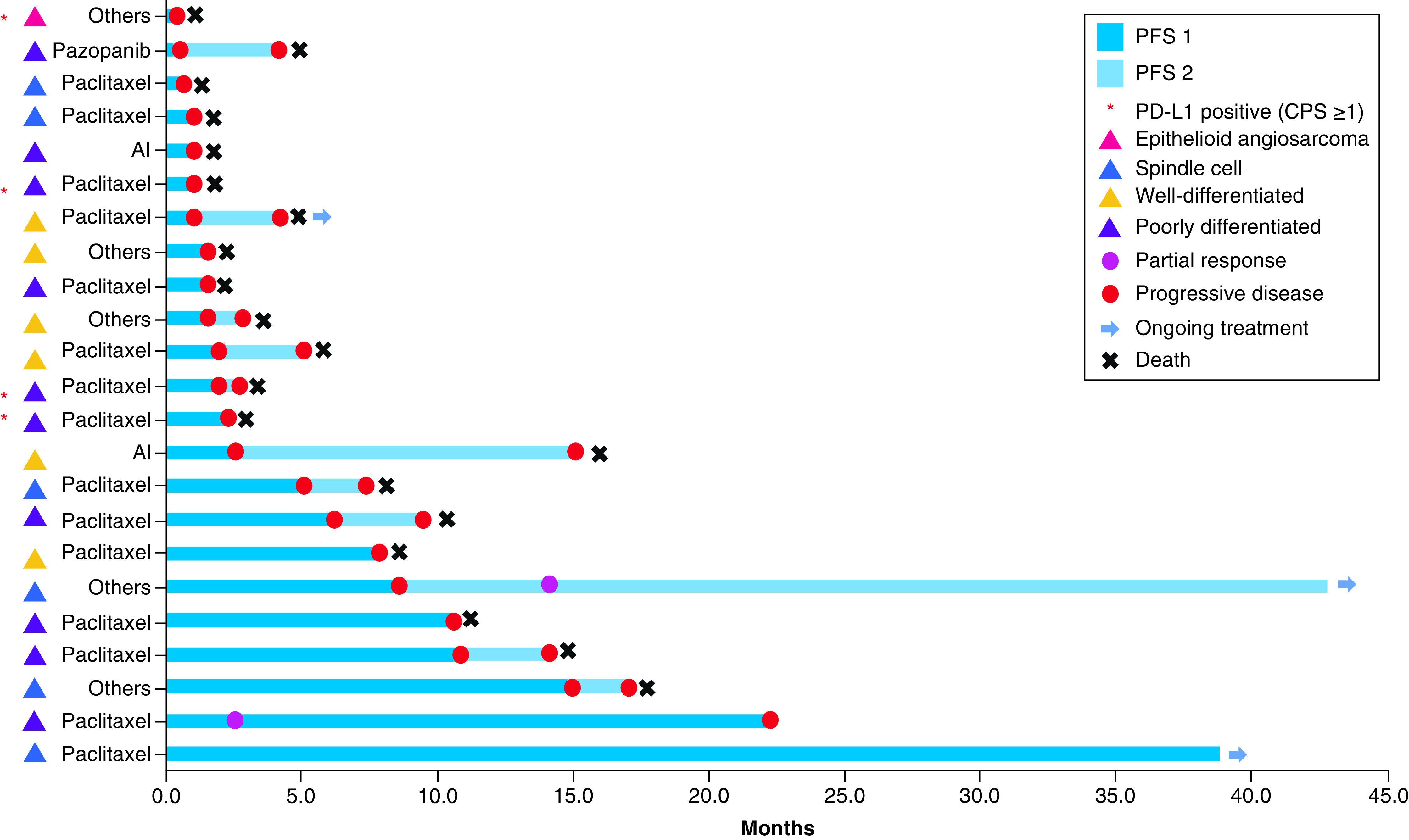
Swimmer plot for initial metastatic angiosarcoma patients treated with palliative chemotherapy (n = 23). Each patient’s initial treatment is described in the left column. Other regimens include ifosfamide (n = 1); doxorubicin and olaparib (n = 1); mesna, doxorubicin, ifosfamide and dacarbazine (n = 1); etoposide, ifosfamide and cisplatin (n = 1); vincristine, cyclophosphamide and doxorubicin alternatively followed by ifosfamide and etoposide (n = 1). AI: Adriamycin and ifosfamide; CPS: Combined proportion score; PD-L1: Programmed death ligand-1; PFS: Progression-free survival.

We explored the correlation between PD-L1 expression (CPS ≥1) and clinicopathological features. Among 70 patients, 13 (19%) were positive for PD-L1 expression. There was a significant difference in histology (p = 0.01) between the PD-L1-positive and -negative groups. In the PD-L1-positive group, nine of 13 patients (19%) were categorized as poorly differentiated. The remaining patients included well-differentiated (n = 1), spindle (n = 1) and epithelioid (n = 3) angiosarcoma. Notably, all three cases of epithelioid angiosarcoma patients showed high levels of PD-L1 expression, with CPS ≥50 (Figure 1). This is in stark contrast to the result in the PD-L1-negative group, which included a considerable number of patients with well-differentiated (n = 28, 97%) and spindle angiosarcomas (n = 13, 93%). Other factors such as sex, tumor location, histology, initial distant metastasis, surgery, resection margin, definitive radiotherapy, adjuvant chemotherapy, adjuvant radiotherapy, palliative chemotherapy, palliative radiotherapy and first-line regimen were not significantly different between the two groups. In addition, we investigated whether clinicopathological features differed according to different CPS cutoffs (10, 50) and tumor proportion score (1, 10, 50) (Supplementary Tables 1 & 2). Among the patients who were both diagnosed initially with metastatic angiosarcoma and received palliative systemic chemotherapy (n = 23), there were four PD-L1-positive patients (Figure 2).

Patients’ PD-L1 expression was positive with CPS of 100, 5, 60 and 90, in the order delineated in the Swimmer plot. One of these patients who was diagnosed with epithelioid angiosarcoma received doxorubicin and olaratumab. Three other patients with poorly differentiated pathology received paclitaxel as the first-line treatment. These patients showed rapid progression. The remaining 19 patients with no PD-L1 expression showed variable responses to first-line chemotherapeutic agents.

### Survival outcome according to PD-L1 expression

**Figure 3.**
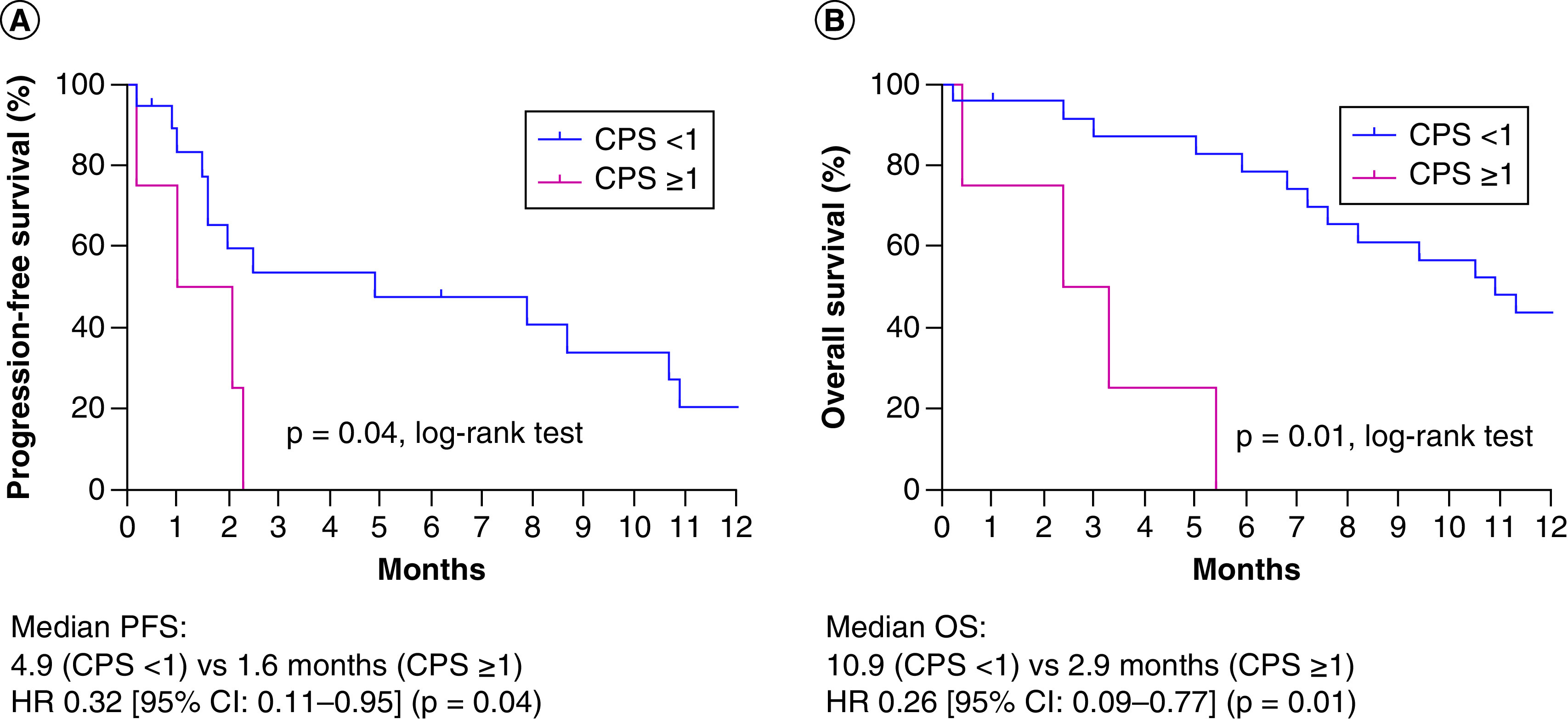
Survival analysis of initial metastatic angiosarcoma patients (n = 28) with combined positive score ≥1. Kaplan–Meier estimates of **(A)** PFS (n = 23) and **(B)** OS (n = 28). CPS: Combined positive score; HR: Hazard ratio; OS: Overall survival; PFS: Progression-free survival.

There was no significant difference in median PFS (mPFS) of both recurrent and metastatic angiosarcoma patients (n = 44) and the mOS of the total patient population (n = 70) (Supplementary Figure 1). We also analyzed the clinical relevance of PD-L1 expression in this subset of patients who received palliative chemotherapy (n = 23) and found that the PD-L1-negative patients (n = 19) had longer mPFS and mOS than PD-L1-positive patients (n = 4) (Figure 3A). The mPFS were 4.9 and 1.6 months (hazard ratio [HR]: 0.32; 95% CI: 0.11–0.95) in PD-L1-negative and PD-L1-positive patients, respectively (p = 0.04). The median OS was 10.9 months for PD-L1-negative patients and 5.4 months (HR: 0.26; 95% CI: 0.09–0.77; p = 0.01) for PD-L1-positive patients (Figure 3B).

**Figure 4.**
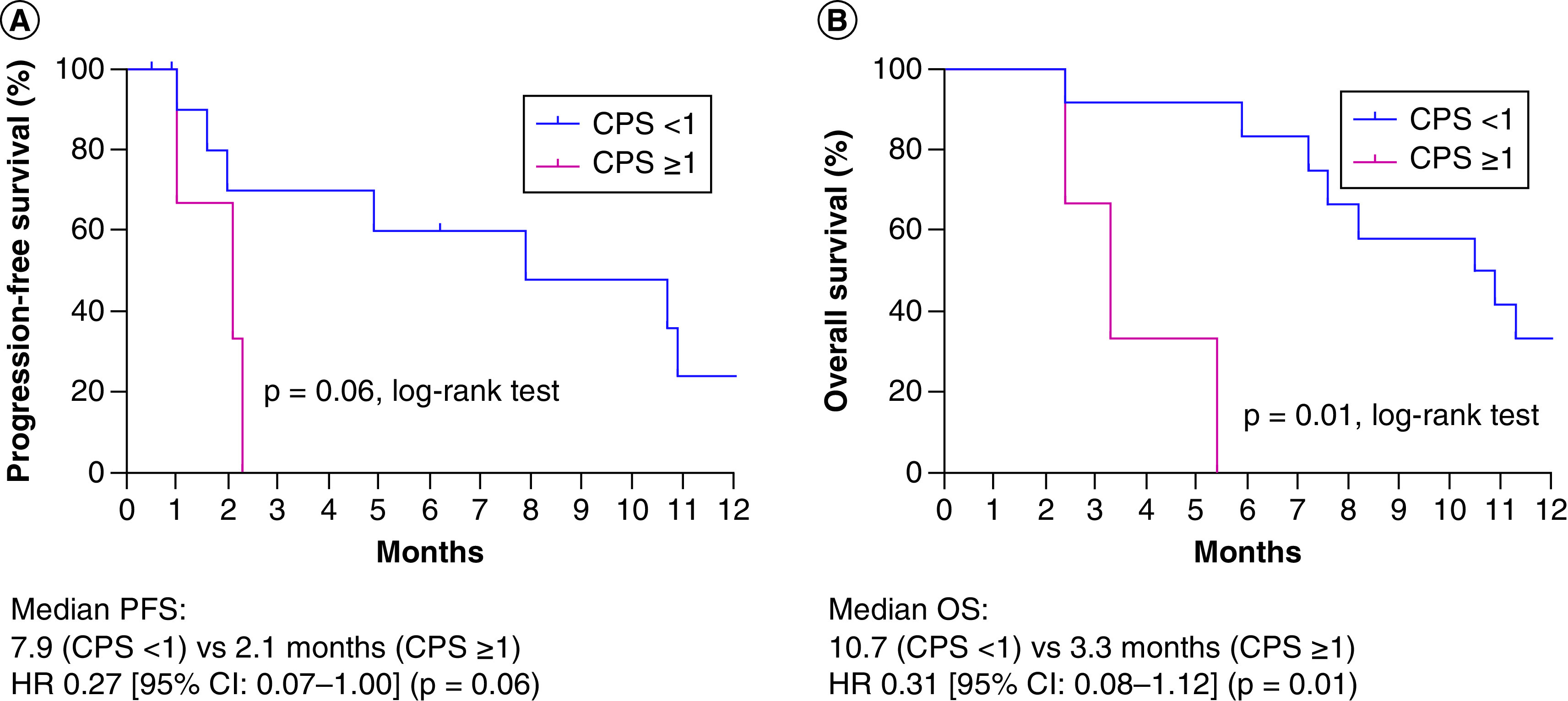
Survival analysis of initial metastatic angiosarcoma patients treated with paclitaxel with a combined positive score of 1 (n = 12) or higher (n = 3). Kaplan–Meier estimates of **(A)** PFS and **(B)** OS. CPS: Combined positive score; HR: Hazard ratio; OS: Overall survival; PFS: Progression-free survival.

Regarding treatment efficacy, we further analyzed the prognostic value of PD-L1 expression for patients who received paclitaxel as first-line palliative treatment. Among the 15 cases, PD-L1 expression was positive in 12 patients (80%) and negative in three patients (20%). mPFS were 7.9 and 2.1 months in the PD-L1-negative and PD-L1-positive groups, respectively (HR: 0.27; 95% CI: 0.07–1.00; p = 0.06) (Figure 4A). Although there was a trend for longer PFS in the PD-L1-negative angiosarcoma patients treated with paclitaxel, the difference was not statistically significant. mOS was 10.7 months for PD-L1-negative patients and 3.3 months for PD-L1-positive patients (HR: 0.31; 95% CI: 0.08–1.12; p = 0.01) (Figure 4B).

In univariate analysis, the PD-L1 status (p = 0.01) and margin status (p = 0.01) were associated with OS in initially metastatic angiosarcoma patients (n = 28) (Table 2). However, sex, age, tumor size, histology, tumor location (axial vs extremity), surgery and palliative chemotherapy were not associated with OS. In multivariate analysis, only the PD-L1 status (HR: 7.62; 95% CI: 1.73–33.5; p = 0.01) remained significant, whereas the margin status was not associated with OS.

**Table 2.** Univariate and multivariate analyses of overall survival in initial metastatic angiosarcoma patients (n = 28).

Variables	Univariate		Multivariate	
	OS (months)	p-value	HR (95% CI)	p-value
Sex (male vs female)	10.9 vs 9.4	0.69		
Age (≥20 vs <20 years)	8.2 vs 10.9	0.27		
Tumor size (≥5 cm vs <5 cm)	7.2 vs 11.3	0.12		
Histology (spindle vs others^†^)	10.5 vs 8.2	0.09		
PD-L1 status (CPS <1 vs ≥1)	2.3 vs 10.9	0.01	7.62 (1.73–33.5)	0.01
Tumor location (axial vs extremity)	9.4 vs 13.6	0.54		
Surgery (yes vs no)	10.9 vs 7.6	0.20		
Margin status (positive vs negative)	Not reached vs 7.6	0.01	0.05 (0–1.25)	0.07
Palliative chemotherapy (yes vs no)	8.2 vs 30	0.33		

† Others: well differentiated (n = 8), poorly differentiated (n = 11), epithelioid (n = 1).

CPS: Combined positive score; HR: Hazard ratio; OS: Overall survival; PD-L1: Programmed death ligand-1.

## Discussion

In our study, we retrospectively examined 70 angiosarcoma patients diagnosed at our cancer center. We selected 28 patients with initial metastatic angiosarcoma, and analyzed the clinical relevance of PD-L1 expression with CPS ≥1. Patients with PD-L1 expressions had worse mPFS and mOS than those with negative PD-L1 expressions. Notably, the patients who progressed in the metastatic setting had strong PD-L1 expression. All three epithelioid angiosarcoma patients had high levels of PD-L1 expression with CPS ≥50. Comprehensive characterization of PD-L1-positive epithelioid angiosarcoma is warranted in a larger cohort to validate whether this particular subset of patients has worse prognosis.

Angiosarcoma is highly aggressive and a rare type of tumor. It is highly underrepresented even within other STSs, and the heterogeneity with different pathologic subtypes and the wide range of clinicopathological and genomic complexities further limit treatment options and opportunities for clinical studies [20]. There are few publications that have addressed the prognostic role of PD-L1 expression in STS [21–28]. These studies were based on immunohistochemistry (IHC) on tissue microarrays or slides, all of which used different scoring systems and definitions of PD-L1-positive expression. Two studies defined PD-L1 expression as PD-L1 detected in ≥5% of tumor cells [24,28]. In a retrospective study of cutaneous angiosarcoma of 52 patients, PD-L1 was defined in semiquantitative manner, with tumors with score >2 (PD-L1: 5–10%) graded as positive [26]. Another study analyzed PD-L1 mRNA expression in 470 patients and defined low and high PD-L1 expression using a receiver operating characteristic curve for determining the cutoff value [23]. Only a few angiosarcoma patients were evaluated in these studies, and the discrepancies and variabilities in the PD-L1 assay made it difficult to determine PD-L1 expression as a prognostic biomarker, especially for the subset of angiosarcoma patients. Overall, results of the role of PD-L1 as prognostic marker in metastatic angiosarcoma were conflicting. Some studies showed that PD-L1 expression was associated with poor prognosis [21,23,26,27], while one study of cutaneous angiosarcoma with PD-L1 expressions had favorable survival in stage I [28]. Other studies concluded that there were no correlations [24,25].

Recently, studies were conducted for assessing the impact of different IHC platforms and the cutoff value for PD-L1 expression in multiple tumors [29,30]. In our study, we used standardized IHC criteria for PD-L1 expression. PD-L1, clone 22C3 (Dako, CA, USA), was used as the primary antibody and defined PD-L1 expression as CPS ≥1. Similar to our results, tumors with high levels of PD-L1 expression have shown worse survival [22,31–33].

Immune evasion is one of the hallmarks of cancer, and PD-L1 is the key immune checkpoints that orchestrates immune escape and prevents antitumor immune responses, especially during the maintenance of peripheral tolerance [34]. Thus, novel therapies such as those targeting the PD-1/PD-L1 axis have gained wide recognition for treating solid tumors to overcome the immune suppressive environment [35]. Currently, the paradigm has shifted from chemotherapy and targeted agents to immunotherapies in several tumors [36,37]. Anti-PD-L1 agents such as pembrolizumab and nivolumab have also been studied in metastatic STS [15,16]. With the exception of that in undifferentiated pleomorphic sarcoma or dedifferentiated liposarcoma, pembrolizumab failed to meet its primary end point of objective response [16]. Nivolumab monotherapy also failed to show clinical benefit in STS patients [15]. Of note, none of the patients with metastatic angiosarcomas were enrolled in either pembrolizumab or nivolumab monotherapy. However, three angiosarcoma patients were enrolled in the nivolumab and ipilimumab combination group, and the mOS of 42 patients with a different histology was 14 months (95% CI: 9.6–not reached), which is comparable to that of patients treated with chemotherapy agents [38,39].

To overcome the limitation of anti-PD-L1 monotherapy, combination treatments with chemotherapy may be a possible option for metastatic STS, including angiosarcoma. Certainly, platinum-based chemotherapy with pembrolizumab has improved the efficacy in non-small-cell lung cancers, and this combination is also being tested for other solid tumors [40–42]. The efficacy of durvalumab with doxorubicin as the first-line treatment in STS is currently under investigation at our center (ClinicalTrials.gov Identifier: NCT03802071).

The main limitations of this study were its use of retrospective data and a small sample size owing to the rarity of angiosarcoma cases. Even though we collected a large cohort of angiosarcoma patients, only 40% were initially diagnosed in the metastatic setting and a third of them received palliative chemotherapies. Since there were only significant outcomes for the initially metastatic patients, we excluded recurrent patients who also received chemotherapies, thus further limiting the sample size for the validation of PD-L1 as a prognostic factor for angiosarcoma. Therefore, the prognostic role of PD-L1 should be investigated in larger angiosarcoma cohorts.

## Conclusion

Patients initially diagnosed with metastatic angiosarcoma expressing PD-L1 by CPS ≥1 had shorter mPFS and mOS, and multivariate analysis showed that PD-L1 is an independent prognostic factor for OS. A larger number of patients should be recruited in a prospective manner for prognostic validation of PD-L1 in metastatic angiosarcoma.

## Future perspective

Metastatic angiosarcoma has poor prognosis, and identifying prognostic markers may provide better treatment options in the future. Retrospective analysis of the patients in metastatic settings with high PD-L1 by CPS ≥1 had shorter OS, thus supporting the validation of PD-L1 as an independent prognostic marker. Our results are stepping stones for future studies exploring the PD-L1 expression as a useful prognostic tool in angiosarcoma.

**Summary points**

- We investigated the feasibility of using programmed death ligand-1 (PD-L1) expression as a prognostic biomarker in metastatic angiosarcoma patients.
- We retrospectively examined 70 angiosarcoma patients who were diagnosed at our center between 2005 and 2019.
- We then selected 28 patients with initial metastatic angiosarcoma to analyze the clinical relevance of PD-L1 expression with combined positive score (CPS) ≥1.
- Among metastatic angiosarcoma patients (n = 28), PD-L1-negative patients (n = 24) showed longer median progression-free survival (4.9 vs 1.6 months; p = 0.04) and median overall survival (10.9 vs 5.4 months; p = 0.01) than PD-L1-positive patients (n = 4).
- Multivariate analysis showed PD-L1 status as a significant factor for overall survival in metastatic angiosarcoma patients.
- Swimmer plot showed that all three epithelioid angiosarcoma patients had high expression levels of PD-L1 with CPS ≥50. Further characterization of PD-L1-positive epithelioid angiosarcoma is warranted in a larger prospective cohort.
- Our findings suggest that the assessment of PD-L1 expression as a diagnostic and therapeutic strategy could be implemented in metastatic angiosarcoma.
