# Supplementary material for: Prognostic implications of PD-L1 expression in patients with angiosarcoma
Source: Future Sci OA. 2021 Mar 2;7(5):FSO691. doi: 10.2144/fsoa-2020-0211 (PMC8147824; doi:10.2144/fsoa-2020-0211)
Supplement: Supplementary file 1 [file fsoa-07-691-s1.pptx]

## Slide 1
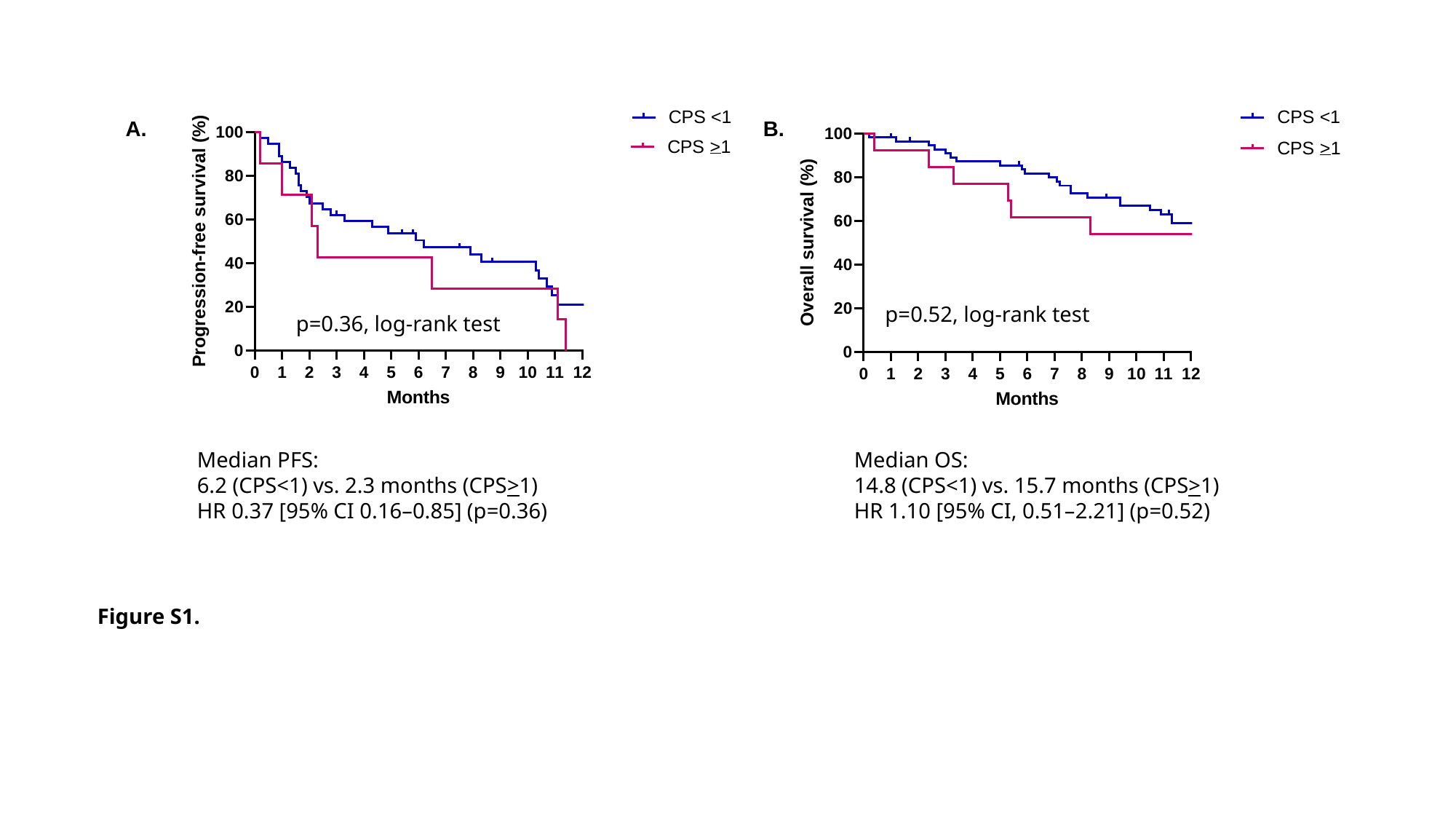

A.
B.
p=0.52, log-rank test
p=0.36, log-rank test
Median PFS:
6.2 (CPS<1) vs. 2.3 months (CPS>1)
HR 0.37 [95% CI 0.16–0.85] (p=0.36)
Median OS:
14.8 (CPS<1) vs. 15.7 months (CPS>1)
HR 1.10 [95% CI, 0.51–2.21] (p=0.52)
Figure S1.
