# Supplementary material for: Prognostic implications of PD-L1 expression in patients with angiosarcoma
Source: Future Sci OA. 2021 Mar 2;7(5):FSO691. doi: 10.2144/fsoa-2020-0211 (PMC8147824; doi:10.2144/fsoa-2020-0211)
Supplement: Supplementary file 2 [file fsoa-07-691-s2.docx]

**Table S1.** Baseline characteristics of subgroups by PD-L1 combined positive score

| **Variables** |  |  | **PD-L1 expression** | | | | | |
| --- | --- | --- | --- | --- | --- | --- | --- | --- |
|  |  | **Total** | **CPS < 10** | **CPS >10** | **p value** | **CPS < 50** | **CPS > 50** | **p value** |
|  |  | **n=70** | **n=61 (87%)** | **n=9 (13%)** |  | **n=65 (92%)** | **n=5 (8%)** |  |
| Sex | Male | 40 | 37 (92%) | 3 (8%) | 0.16 | 37 (92%) | 3 (8%) | 0.99 |
|  | Female | 30 | 24 (80%) | 6 (20%) |  | 24 (80%) | 6 (20%) |  |
| Median age (range) |  | 61 (7-86) | 62 (7-86) | 56 (25-74) |  | 61 (4-86) | 54 (45-74) |  |
| Median tumor size (cm, range) | | 4 (0.3-99) | 4 (0.3-99) | 5.5 (0.7-61) |  | 4 (0.3-99) | 5 (0.7-61) |  |
| Tumor location | Head and neck | 28 | 24 (86%) | 4 (14%) | 0.35 | 26 (93%) | 2 (7%) | 0.23 |
|  | Thorax | 15 | 14 (93%) | 1 (7%) |  | 15 (100%) | 0 (0%) |  |
|  | Abdomen | 18 | 14 (78%) | 4 (22%) |  | 15 (83%) | 3 (17%) |  |
|  | Extremity | 9 | 9 (100%) | 0 (0%) |  | 9 (100%) | 0 (0%) |  |
| Histology | Well differentiated | 29 | 28 (97%) | 1 (3%) | 0.01 | 29 (100%) | 0 (0%) | 0.01 |
|  | Spindle | 14 | 13 (93%) | 1 (7%) |  | 14 (100%) | 0 (0%) |  |
|  | Epithelioid | 3 | 0 (0%) | 3 (100%) |  | 0 (0%) | 3 (100%) |  |
|  | Poorly differentiated | 24 | 20 (83%) | 4 (17%) |  | 22 (92%) | 2 (8%) |  |
| Initial distant metastasis | No | 42 | 36 (86%) | 6 (14%) | 0.73 | 40 (95%) | 2 (5%) | 0.38 |
|  | Yes | 28 | 25(89%) | 3 (11%) |  | 25 (89%) | 3 (11%) |  |
| Surgery | No | 29 | 26 (90%) | 3 (10%) | 0.73 | 26 (90%) | 3 (10%) | 0.64 |
|  | Yes | 41 | 35 (85%) | 6 (15%) |  | 39 (95%) | 2 (5%) |  |
| Resection margin | Negative | 43 | 37 (86%) | 6 (14%) | 0.41 | 38 (88%) | 5 (12%) | 0.16 |
|  | Positive | 21 | 20 (95%) | 1 (5%) |  | 21 (100%) | 0 (0%) |  |
| Definitive radiotherapy | No | 59 | 51 (86%) | 8 (14%) | 0.99 | 55 (93%) | 4 (7%) | 0.99 |
|  | Yes | 11 | 10 (91%) | 1 (9%) |  | 10 (90%) | 1 (10%) |  |
| Adjuvant chemotherapy | No | 64 | 56 (97%) | 8 (13%) | 0.58 | 59 (92%) | 5 (8%) | 0.99 |
|  | Yes | 6 | 5 (83%) | 1 (17%) |  | 6 (100%) | 0 (0%) |  |
| Adjuvant radiotherapy | No | 51 | 46 (90%) | 5 (10%) | 0.24 | 47 (92%) | 4 (8%) | 0.99 |
|  | Yes | 19 | 15 (79%) | 4 (21%) |  | 18 (95%) | 1 (5%) |  |
| Palliative chemotherapy | No | 26 | 21 (81%) | 5 (19%) | 0.28 | 24 (92%) | 2 (8%) | 0.99 |
|  | Yes | 44 | 40 (91%) | 4 (9%) |  | 41 (93%) | 3 (7%) |  |
| Palliative radiotherapy | No | 43 | 36 (84%) | 7 (16%) | 0.47 | 40 (93%) | 3 (7%) | 0.99 |
|  | Yes | 27 | 25 (93%) | 2 (7%) |  | 25 (93%) | 2 (7%) |  |
| First-line chemotherapy | Paclitaxel | 31 | 29 (94%) | 2 (6%) | 0.37 | 29 (94%) | 2 (6%) | 0.82 |
|  | Pazopanib | 3 | 3 (100%) | 0 (0%) |  | 3 (100%) | 0 (0%) |  |
|  | Others | 10 | 8 (80%) | 2 (20%) |  | 9 (90%) | 1 (10%) |  |

**Abbreviations:** PD-L1, programmed death-ligand 1; CPS, combined positive score

**Table S2.** Baseline characteristics of subgroups by PD-L1 tumor proportion score

| **Variables** |  |  | **PD-L1 expression** | | | **PD-L1 expression** | | | | | |
| --- | --- | --- | --- | --- | --- | --- | --- | --- | --- | --- | --- |
|  |  | **total** | **TPS <1** | **TPS >1** | **p value** | **TPS < 10** | **TPS >10** | **p value** | **TPS < 50** | **TPS >50** | **p value** |
|  |  | **n=70 (%)** | **n= 62 (89%)** | **n=8 (11%)** |  | **n=63 (90%)** | **n=7 (10%)** |  | **n=65 (92%)** | **n=5 (8%)** |  |
| Sex | Male | 40 | 37 (92%) | 3 (8%) | 0.27 | 37 (92%) | 3 (8%) | 0.45 | 37 (92%) | 3 (8%) | 0.99 |
|  | Female | 30 | 25 (83%) | 5 (17%) |  | 26 (87%) | 4 (13%) |  | 24 (80%) | 6 (20%) |  |
| Median age (range) | | 61 (7-86) | 57 (7-86) | 57 (45-74) |  | 62 (7-86) | 56 (45-74) |  | 61 (4-86) | 54 (45-74) |  |
| Median tumor size (cm, range) | | 4 (0.3-99) | 4 (0.3-99) | 5 (0.7-61) |  | 4 (0.3-99) | 5.5 (0.7-61) |  | 4 (0.3-99) | 5 (0.7-61) |  |
| Tumor location | Head and neck | 28 | 24 (86%) | 4 (14%) | 0.14 | 25 (89%) | 3 (11%) | 0.13 | 26 (93%) | 2 (7%) | 0.23 |
|  | Thorax | 15 | 15 (100%) | 0 (0%) |  | 15 (100%) | 0 (0%) |  | 15 (100%) | 0 (0%) |  |
|  | Abdomen | 18 | 14 (78%) | 4 (22%) |  | 14 (78%) | 4 (22%) |  | 15 (83%) | 3 (17%) |  |
|  | Extremity | 9 | 9 (100%) | 0 (0%) |  | 9 (100%) | 0 (0%) |  | 9 (100%) | 0 (0%) |  |
| Histology | Well differentiated | 29 | 29 (100%) | 0 (0%) | 0.01 | 29 (100%) | 0 (0%) | 0.01 | 29 (100%) | 0 (0%) | 0.01 |
|  | Spindle | 14 | 13 (93%) | 1 (7%) |  | 13 (92%) | 1 (8%) |  | 14 (100%) | 0 (0%) |  |
|  | Epithelioid | 3 | 0 (100%) | 3 (100%) |  | 0 (100%) | 3 (100%) |  | 0 (0%) | 3 (100%) |  |
|  | Poorly differentiated | 24 | 20 (83%) | 4 (17%) |  | 21 (87%) | 3 (13%) |  | 22 (92%) | 2 (8%) |  |
| Initial distant metastasis | No | 42 | 37 (88%) | 5 (12%) | 0.99 | 38 (90%) | 4 (10%) | 0.99 | 40 (95%) | 2 (5%) | 0.38 |
|  | Yes | 28 | 25 (89%) | 3 (11%) |  | 25 (89%) | 3 (11%) |  | 25 (89%) | 3 (11%) |  |
| Surgery | No | 29 | 26 (90%) | 3 (10%) | 0.99 | 26 (90%) | 3 (10%) | 0.99 | 26 (90%) | 3 (10%) | 0.64 |
|  | Yes | 41 | 36 (88%) | 5 (12%) |  | 37 (90%) | 4 (10%) |  | 39 (95%) | 2 (5%) |  |
| Resection margin | Negative | 43 | 38 (88%) | 5 (12%) | 0.65 | 38 (88%) | 5 (12%) | 0.99 | 38 (88%) | 5 (12%) | 0.16 |
|  | Positive | 21 | 20 (95%) | 1 (5%) |  | 21 (100%) | 0 (0%) |  | 21 (100%) | 0 (0%) |  |
| Definitive radiotherapy | No | 59 | 53 (90%) | 6 (10%) | 0.6 | 53 (90%) | 6 (10%) | 0.99 | 55 (93%) | 4 (7%) | 0.99 |
|  | Yes | 11 | 9 (82%) | 2 (18%) |  | 10 (91%) | 1 (9%) |  | 10 (90%) | 1 (10%) |  |
| Adjuvant chemotherapy | No | 64 | 57 (89%) | 7 (11%) | 0.53 | 58 (91%) | 6 (9%) | 0.48 | 59 (92%) | 5 (8%) | 0.99 |
|  | Yes | 6 | 5 (83%) | 1 (17%) |  | 5 (83%) | 1 (17%) |  | 6 (100%) | 0 (0%) |  |
| Adjuvant radiotherapy | No | 51 | 46 (90%) | 5 (10%) | 0.68 | 43 (92%) | 4 (8%) | 0.4 | 47 (92%) | 4 (8%) | 0.99 |
|  | Yes | 19 | 16 (84%) | 3 (16%) |  | 16 (84%) | 3 (16%) |  | 18 (95%) | 1 (5%) |  |
| Palliative chemotherapy | No | 26 | 22 (85%) | 4 (15%) | 0.46 | 22 (85%) | 4 (15%) | 0.41 | 24 (92%) | 2 (8%) | 0.99 |
|  | Yes | 44 | 40 (91%) | 4 (9%) |  | 41 (93%) | 3 (7%) |  | 41 (93%) | 3 (7%) |  |
| Palliative radiotherapy | No | 26 | 22 (85%) | 4 (15%) | 0.42 | 38 (888%) | 5 (12%) | 0.7 | 40 (93%) | 3 (7%) | 0.99 |
|  | Yes | 27 | 25 (93%) | 2 (7%) |  | 25 (93%) | 2 (7%) |  | 25 (93%) | 2 (7%) |  |
| First line chemotherapy | Paclitaxel | 31 | 28 (90%) | 3 (10%) | 0.85 | 28 (90%) | 3 (10%) | 0.82 | 29 (94%) | 2 (6%) | 0.82 |
|  | Pazopanib | 3 | 3 (100%) | 0 (0%) |  | 3 (100%) | 0 (0%) |  | 3 (100%) | 0 (0%) |  |
|  | Others | 10 | 9 (90%) | 1 (10%) |  | 9 (90%) | 1 (10%) |  | 9 (90%) | 1 (10%) |  |

**Abbreviations:** PD-L1, programmed death-ligand 1; TPS, tumor proportion score
